# Synthesis and Anti-Influenza Virus Activities of a Novel Class of Gastrodin Derivatives

**DOI:** 10.3390/molecules18043789

**Published:** 2013-03-26

**Authors:** Si-Tu Xue, Wei-Ying He, Lin-Lin Ma, Hui-Qiang Wang, Bo Wang, Guang-Hui Zheng, Xing-Yue Ji, Tian Zhang, Yu-Huan Li, Jian-Dong Jiang, Zhuo-Rong Li

**Affiliations:** Institute of Medicinal Biotechnology, Chinese Academy of Medical Sciences & Peking Union Medical College, Beijing 100050, China

**Keywords:** gastrodin, aryl glycoside, synthesis, influenza virus inhibitors

## Abstract

A series of substituted aryl glycoside analogues of gastrodin have been identified as potential anti-influenza agents. The most potent inhibitor **1a** exhibited moderate inhibitory activity against the A/Hanfang/359/95(H3N2) and A/FM/1/47(H1N1) strains of the influenza A virus (IC_50_ values of 44.40 and 34.45 μM, respectively) and the oseltamivir-null B/Jifang/13/97 strain of influenza B (IC_50_ value of 33.01 μM). In this article, multiple doses of compound **1a** (80 mg/kg/day, oral administration) were used for the treatment of mice infected with influenza A/FM/1/47-MA (H1N1), and surprisingly we found that compound **1a** significantly increased the number of survivors and prolonged the mean survival time. The preliminary studies on the mechanism of antiviral activity showed no interaction between compound **1a** and the neuraminidase or the M2 protein. The novel target to overcome drug resistance combined with its good *in vivo* profile support compound **1a** to be a new lead for further development of antiviral agents.

## 1. Introduction

Influenza is a respiratory disease caused by the influenza virus. According to the World Health Organization (WHO), as of April 4, 2010, the 2009-H1N1 influenza A virus have spread to over 213 countries and resulted in over 600,000 laboratory-confirmed cases and at least 17,700 deaths (WHO, 2010). These numbers represent the tip of the iceberg with regard to the potential morbidity and mortality associated with infection resulting from this virus [1].

**Figure 1:**
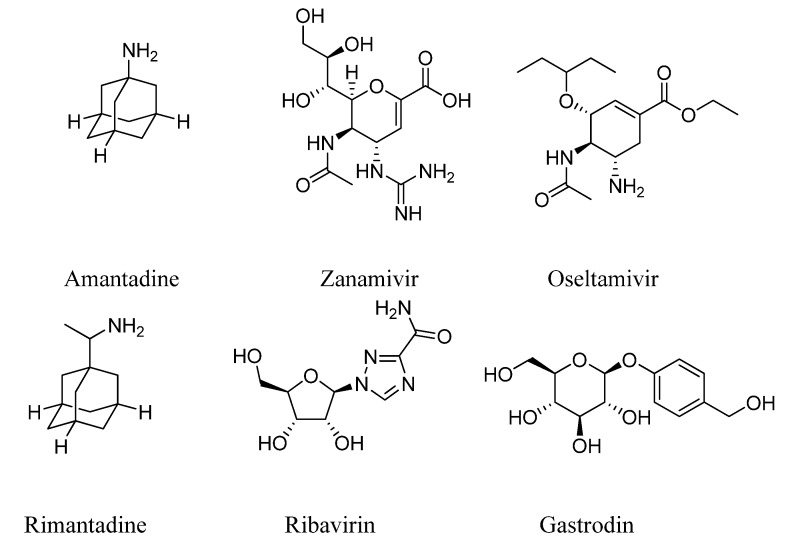
The chemical structures of some anti-influenza drugs and gastrodin.

Despite the extensive effort has been invested in attempting to control influenza infection, only two classes of drugs are currently clinically available, namely inhibitors of the M2 protein (e.g., amantadine and rimantadine) and the neuraminidase (e.g., zanamivir and oseltamivir) (Figure 1). Clinical applications of amantadine and rimantadine have been limited as a consequence of the increasing incidence of adamantane-resistant viruses in the general population [2,3]. Moreover, blockers of the M2 ion channel inhibit only the replication of influenza A virus and could cause neurological side effects [4]. The neuraminidase inhibitors, zanamivir and oseltamivirwere marketed in 1999 for the treatment and prophylaxis of influenza and have been subsequently stockpiled by many countries for usage in the event of a pandemic. However, significant increases in the frequency of oseltamivir-resistant seasonal influenza A (H1N1) in Europe, the United States, Oceania and South Africa have been identified recently. By April 30, 2010, a total of 285 cases of resistance had been reported worldwide [5], and the widespread emergence of oseltamivir-resistant strains had become increasingly likely [6]. In addition to mutation of H275, an S274N neuraminidase mutation has also been reported [7]. The recent pandemic of influenza A (H1N1) effectively reemphasizes the importance of identifying new anti-influenza drugs. 

*Gastrodia elata* Blume, which is an orchid plant, is a well-known Traditional Chinese Medicine and has been clinically used for about 2,000 years. Its medicinal part is the dry tuber, and it is widely beleived that as a traditional medicine *Gastrodia elata* Blume has anti-epileptic, anti-palpitation, anti-rheumatic, tranquil and nourishing effect. Gastrodin, which was isolated and identified from the extracts of *Gastrodia elata* Blume, has been widely used in the clinic in China for the treatment of hypertension, headache and neuralgia [8]. To the best of our knowledge, it has never been used as an antiviral agent in clinic, and there are also no reports about its possible antiviral activities.

In the current work, a series of substituted aryl glycosides were synthesized as analogues of gastrodin and their anti-influenza activity was evaluated. The compounds proved to be a novel class of potential anti-influenza agents. The most potent compound [methyl 4-fluoro-3-((2*S*,3*R*,4*S*,5*R*,6*R*)-3,4,5-triacetoxy-6-(acetoxymethyl)-tetrahydro-2*H*-pyran-2-yloxy) benzoate (**1a**)] exhibited significant inhibitory activity against the tested influenza A strains: A/Hanfang/359/95(H3N2) and A/FM/1/47(H1N1), with IC_50_ values of 44.4 and 34.4 μM, respectively. Mice infected with influenza A/FM/1/47-MA (H1N1) were treated with multiple doses of compound **1a** on a dose regimen of 80 mg/kg/day (oral administration), which led to a significant increase in the number of survivors and a prolonged mean survival time. Herein, we reported the synthesis and biological evaluation of a series of novel gastrodin analogues as new antiviral agents for the first time, along with a discussion of the preliminary structure-activity relationships (SAR) study and the *in vivo* activity.

## 2. Results and Discussion

### 2.1. Chemistry

The chemical structures of the target analogues differed in three ways, and the modifications are as follows: (1) changes to the substituents and their positions on the phenyl ring; (2) conversion of the phenyl ring to a pyridyl ring; and (3) changes to the sugar moiety or protection of the hydroxyl group by acetylation.

**Scheme 1:**
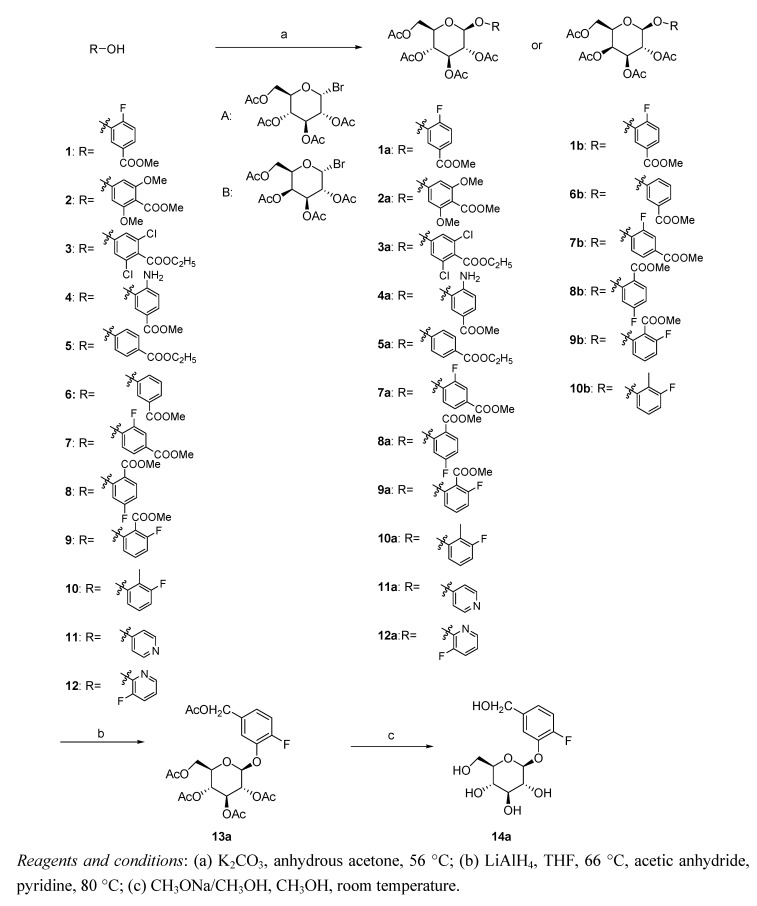
The synthetic route to the target compounds.

As shown in the Scheme 1, compound **1a** was synthesized from the reaction of methyl 4-fluoro-3-hydroxybenzoate (**1**) with 2,3,4,6-tetra-*O*-acetyl-α-d-glucopyranosyl bromide using potassium carbonate (K_2_CO_3_) as the base. The color of the reaction system darkened slightly as the reaction proceeded. Unfortunately, we were unable to develop conditions for driving the reaction to completion. Consequently, significant levels of the starting material were detected in the reaction mixture, and a low yield of less than 40% was obtained. Compound **13a** was obtained from **1a** in a two-step procedure involving the initial reduction of the methyl ester in **1a** with lithium aluminum hydride (LiAlH_4_) [9] followed by acetylation of the resulting primary alcohol with acetic anhydride. Subsequent deprotection of the acetyl groups by saponification with sodium methoxide (CH_3_ONa) in MeOH gave **14a** [10] in greater than 70% overall yield from **1a** (Scheme 1). Compounds **2a**–**5a**, **7a**–**12a**, **1b**, **6b**–**10b** were also synthesized by this route, as depicted in Scheme 1.

### 2.2. Evaluation of Anti-Influenzavirus Activity *In Vitro*

The antiviral activities of the synthesized glycoside compounds against influenza A/Hanfang/359/95(H3N2) and A/FM/1/47(H1N1) were initially evaluated through their ability to prevent cytopathic effects (CPE) in influenza A virus-infected Madin-Darby canine kidney (MDCK) cells, with oseltamivir and ribavirin (RBV) as the positive controls. Except for the known compounds, **1** (the methyl 3-hydroxy-4-fluorobenzoate starting material), **5a**, **7a**, **11a**, **6b** and gastrodin, all compounds tested in the current study were, to the best of our knowledge, synthesized and were reported for the first time in academia. Ultimately, a total of 21 compounds were evaluated, and the results were summarized in Table 1.

As shown in Table 1, compounds **1a**, **8a**, **11a** and **10b** exhibited moderate inhibitory activity against the A/Hanfang/359/95(H3N2) strain of the influenza virus, with IC_50_ values of 44.40, 44.40, 40.52 and 48.68 μM, respectively. Nevertheless, they were not as potent as the reference drugs ribavirin (IC_50_ = 11.87 μM) and oseltamivir (IC_50_ = 2.08 μM). Of all the tested compounds, **2a** showed the highest level of inhibitory activity against the A/FM/1/47(H1N1) strain of the influenza virus, with an IC_50_ value of 11.58 μM, which was close to that of the reference drug oseltamivir (IC_50_ value of 57.52 μM). Furthermore, compounds **1a**, **9a**, **11a** and **12a** also exhibited moderate levels of inhibitory activity against the A/FM/1/47(H1N1) strain of the influenza virus, with IC_50_ values of 34.45, 34.45, 52.23 and 25.24 μM, respectively. Compounds **1a** and **11a** showed activity against both the A/Hanfang/359/95(H3N2) and A/FM/1/47(H1N1) strains of the influenza virus. Compounds **5a** and **1b** showed low levels of inhibitory activity against the A/Hanfang/359/95(H3N2) strain of the influenza virus, with IC_50_ values of 93.64 and 92.38 μM, respectively. Compounds **8a** and **9b** showed low levels of inhibitory activity against the A/FM/1/47(H1N1) strain of the influenza virus, with IC_50_ values of 83.72 and 66.56 μM, respectively. In contrast, the remaining compounds (including compound **1** and gastrodin) showed weak or no activity against these strains under the specific conditions used in this paper.

**Table 1:** Anti-influenza activities of the synthesized compounds.

Compounds	TC_50_ (μM)	A/Hanfang/359/95 ^a^		A/FM/1/47 ^b^	
		IC_50_ (μM)	SI	IC_50_ (μM)	SI
**1a**	192.14	44.40	4.3	34.45	5.6
**2a**	23.65	>13.66	_	11.58	2.0
**3a**	>362.77	>362.77	_	>362.77	_
**4a**	>402.05	>134.02	_	119.03	>3.4
**5a**	232.53	93.64	2.5	>134.29	_
**7a**	277.10	103.39	2.5	>133.23	_
**8a**	277.10	44.40	6.2	83.72	3.3
**9a**	>399.66	>44.40	_	34.45	>11.6
**10a**	>438.20	>146.07	_	>146.07	_
**11a**	226.03	40.52	5.6	52.23	4.3
**12a**	350.06	>50.12	_	25.24	13.9
**13a**	>388.76	>129.60	_	>129.60	_
**14a**	>657.31	>657.31	_	>657.31	_
**1b**	>399.66	92.38	2.5	>133.23	_
**6b**	>414.56	>138.19	_	>138.19	_
**7b**	>399.66	>133.22	_	133.22	>3.0
**8b**	>399.66	>399.66	_	>399.66	_
**9b**	399.66	>133.22	_	66.56	6.0
**10b**	252.99	48.68	5.2	>52.23	_
**1**	>1175.50	>1175.50	_	>391.85	_
**Gastrodin**	>698.62	161.49	4.3	>232.19	_
**Ribavirin**	4766.99	11.87	401.4	13.47	353.8
**Oseltamivir**	4033.29	2.08	1938.5	57.52	70.1

^a^ 316TCID_50_; ^b^ 280TCID_50_; Half maximal (50%) inhibitory concentration (IC) of a substance, TC_50_ represents for median lethal dose, and SI represents for Selective therapeutic index.

The active compounds against the A/Hanfang/359/95(H3N2) and A/FM/1/47(H1N1) strains of the influenza virus were further evaluated for their potential antiviral activity against an oseltamivir-null strain of the influenza B virus, also with the means of CPE method. As summarized in Table 2, compounds **1a**, **2a** and **10b** also displayed antiviral activity against the influenza B oseltamivir-null strain. Especially, compound **2a** was found to be the most effective compound against the oseltamivir-resistant strain of the influenza virus (B/Jifang/13/97), with IC_50_ and SI values of 6.25 μM and 6.80, respectively.

**Table 2:** Activities of the synthesized compounds against the oseltamivir-null influenza strain.

Compounds	TC_50_ (μM)	B/Jifang/13/97 ^a^	
		IC_50_ (μM)	SI
**1a**	202.75	33.01	6.14
**2a**	24.63	6.25	3.94
**9a**	399.66	>44.40	_
**11a**	235.95	>52.23	_
**12a**	322.63	>50.12	_
**9b**	360.25	>133.22	_
**10b**	266.16	39.41	6.80
**Ribavirin**	4921.94	11.38	432.35
**Oseltamivir**	3968.32	>100	_

^a^ 100 TCID_50_.

### 2.3. Structure-Antiviral Activity Relationships

As shown in Table 1, compounds **1a**, **8a** and **11a** showed moderate activity against both the A/Hanfang/359/95(H3N2) and A/FM/1/47(H1N1) strains of the influenza virus, whereas compound **10b** only showed moderate activity against the A/Hanfang/359/95(H3N2) strain but no activity against the A/FM/1/47(H1N1) strain. In contrast, compounds **2a**, **9a** and **12a** only showed activity against the A/FM/1/47(H1N1) strain, but no activity against the A/Hanfang/359/95(H3N2) strain. However, all of our active compounds were less potent than the positive controls.

Relative to compound **1a**, the corresponding starting material **1**, which does not contain the 2,3,4,6-tetra-*O*-acetyl-α-d-glucopyranosyl moiety, showed no activity against either of the viral strains. Additionally, when the 2,3,4,6-tetra-*O*-acetyl-α-d-glucopyranosyl moiety was changed to the 2,3,4,6-tetra-*O*-acetyl-α-d-galactopyranosyl moiety, the antiviral activity decreased in the majority of the derivatives and even disappeared in some cases, with only compound **10b** retaining activity against the A/Hanfang/359/95(H3N2). Those results indicated that the 2,3,4,6-tetra-*O*-acetyl-α-d-glucopyranosyl moiety was crucial to the antiviral activity of these compounds. When the fluorine substituent in compound **1a** was replaced with an amino group (compound **4a**), the ester group in compound **1a** was replaced with an acetoxymethyl (compound **13a**) or hydroxymethyl (compound **14a**) group, or the ester group was migrated to the 4-position of the benzene ring (compound **7a**), the antiviral activity was significantly reduced or even disappeared completely in some cases. Interestingly, when the positions of the fluorine and ester groups in compound **1a** were exchanged with each other (compound **8a**), the activity was retained. Taken together, it was possible that possessing hydrophobic substituents (–F, –COOMe) both at the 2- and 5-positions simultaneously enhanced the antiviral activity, yet this need to be confirmed by further experiment. Furthermore, it was also indicated that the presence of a nitrogen-containing heteroaryl ring enhances antiviral activity because compounds **11a** and **12a** both showed antiviral activity. In summary, this is just our preliminary SAR study, and the detailed structure optimization is ongoing in our lab. 

### 2.4. Anti-Influenza Activity of Compound ***1a** in Vivo*

We proceeded to investigate the effect of compound **1a** on influenza A/FM/1/47-MA (H1N1) virus-infected mice, with ribavirin being used as a reference standard. Two doses of **1a** (80 and 40 mg/kg/day) were administered intragastrically. The control compound ribavirin was introduced as a single dose (100 mg/kg/day, po). All experiments were administered twice daily over a seven day period.

Against infection of the influenza A/FM/1/47-MA (H1N1) virus, the ribavirin treatment group exhibited significant increases in mean survival time and moderate decreases in mean lung score. In the group of mice receiving the intragastrically administered dose of 80 mg/kg/day of **1a**, the numbers of surviving animals (6/10) and the mean survival time (11.3 days) increased significantly, whereas all of the control animals died. By contrast, in the group of mice receiving the lower dosage of 40 mg/kg/day of **1a**, only one animal survived (1/10), and the mean survival time was therefore not improved. Therefore, the intragastricl administration of **1a** provided protection against the lethal effects of the influenza A/FM/1/47-MA (H1N1) virus in a dose-dependent fashion.

The effects of **1a** on lung score and on decreasing lung consolidation at day 6 are shown in Table 3. An 80 mg/kg/day dose of **1a** provided a clear improvement in both the lung consolidation and mean lung score relative to that of the vehicle.

**Table 3:** Effects of oral treatment ^a^ with **1a** on influenza A/FM/1/47-MA (H1N1) virus infections ^b^ in mice.

Compound	Dosage (mg/kg/day)	Treated mice			
		% Survivors	Mean day to death ^c^	Lung consolidation ^d^	Mean lung score
**1a**	80 (po)	60 (6/10)	11.3	2.25	1.82 ± 0.82
	40 (po)	10 (1/10)	8.0	3.56	2.61 ± 0.85
**Ribavirin**	100 (po)	100 (10/10)	14.0 *	0.50 *	1.31 ± 0.34 *
**Vehicle**		10 (1/10)	9.0	3.50	2.65 ± 1.06
**Normal controls**		100 (10/10)	14.0 *	0.00 *	1.01 ± 0.12 *

^a^ b.i.d × 7, beginning 1 day before viral infection; ^b^ The viral infection dose was 16.32 LD_50_; ^c^ Mean survival time of mice dying on or before day 14; ^d^ Lungs were removed on day 6 post-virus exposure. Lung scores were determined based on the percentage of the lung displaying signs of consolidation, with 0 being normal and 4 indicative of 100% consolidation. * *p* < 0.05.

### 2.5. The Preliminary Studies on Anti-Influenza Mechanism of Compound ***1a***

Because compound **1a** was orally active and showed potential to be developed as antiviral drug, we researched on anti-influenza mechanism of compound **1a** by neuraminidase inhibition assay, hemagglutination assay and M2 protein expression inhibition assay.

#### 2.5.1. Hemagglutination Inhibition Assay

**Figure 2:**
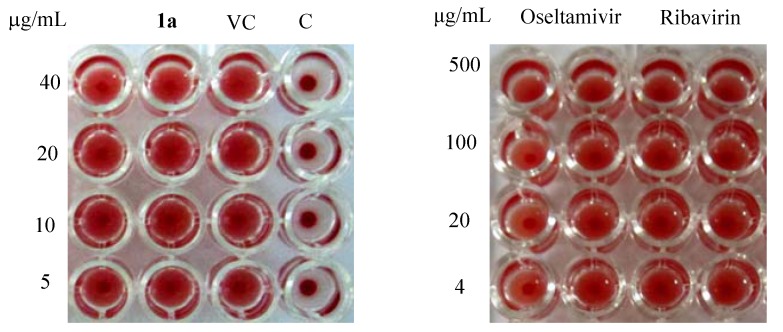
Hemagglutination inhibitory activity of **1a**.

The early influenza virus replication steps include virus absorption, internalization of virus to form a virus-containing endosome, and fusion. Hemagglutination-inhibition test was used in which aggregation of chicken erythrocytes was the consequence of the binding between viral hemagglutinin and sialic acid receptors on the surface of the cells [11]. At 2 fold serial diluted compound **1a** (from 40 μg/mL) (Figure 2), the compound **1a** did not show an inhibitory effect on hemagglutination (data not shown), suggesting that these compounds do not interfere with virus-receptor interaction.

#### 2.5.2. Neuraminidase Inhibition Assay

Neuraminidase inhibition assay were evaluated for *in vitro* inhibitory actions using the method according to the reference with slight modification [12]. This spectrofluorometric assay (excitation wavelength: 355 nm and emission wavelength: 460 nm) uses 2′-(4-methylumbelliferyl)-α-d-acetylneuraminic acid (MUNANA) as the enzyme substrate, which is converted to a fluorescent product upon cleavage by NA. The intensity of fluorescence can reflect the activity of NA sensitively.

The compound **1a** did not have inhibitory effect (IC_50_ > 40 μg/mL) on neuraminidase of the two influenza virus A strain. Oseltamivir has a strong inhibitory effect (IC_50_ = 0.02 μg/mL) on neuraminidase of influenza virus A/Wuhan/359/95.

#### 2.5.3. M2 Protein Expression Inhibition Assay

**Figure 3:**
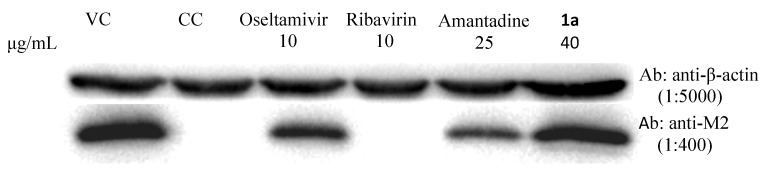
The effect of 1a on M2 protein expression in MDCK cell infected with influenza virus A/Wuhan/359/95.

The effect of compounds on M2 protein expression level were detected by immunoboltting assay [13]. The compound **1a** had no effect on influenza virus M2 protein expression (Figure 3). Amantadine at concentration of 25 μg/mL significantly inhibited influenza virus M2 protein expression. Oseltamivir at concentration of 10 μg/mL has inhibitory effect on influenza virus M2 protein expression, but not so good as amantadine (25 μg/mL). Ribavirin at concentration of 10 μg/mL has significant inhibitory effect on influenza virus M2 protein expression, because at concentration of 10 μg/mL, ribavirin completely inhibits influenza virus induced cytopathic effect.

In summary, compound **1a** tested in hemagglutination inhibition assay, neuraminidase inhibition assay and M2 protein expression inhibition assay have no effect on the three antiviral target of influenza virus. Although this series novel compounds were not as potent as reference compounds, but they had different mechanism against influenza virus. With these results in hand, the future research became more meaningful. Further mechanism studies are now in progress. 

## 3. Experimental

### 3.1. Chemistry

^1^H-NMR spectra were recorded in CDCl_3_ on a 400 or 500 MHz Varian Inova spectrometer. Chemical shifts have been reported in parts per million (ppm) relative to tetramethylsilane (TMS) as the internal standard. The melting points were determined with an X6 microscope melting point apparatus and have been reported uncorrected. Electrospray ionization (ESI) mass spectra and high-resolution mass spectra (HRMS) were obtained on an MDS SCIEX Q-Trap mass spectrometer.

### 3.2. Synthesis

*Methyl 4-fluoro-3-((2S,3R,4S,5R,6R)-3,4,5-triacetoxy-6-(acetoxymethyl)-tetrahydro-2H-pyran-2-yloxy)benzoate* (**1a**). Methyl 4-fluoro-3-hydroxybenzoate (**1**, 1.7 g, 10 mmol), 2,3,4,6-tetra-*O*-acetyl-α-d-glucopyranosyl bromide (5 g, 12 mmol) and K_2_CO_3_ (1.7 g, 12 mmol) were added to anhydrous acetone (75 mL), and the resulting mixture was stirred at 56 °C for 16 h before being cooled to ambient temperature and filtered. The solvent was then evaporated *in vacuo* to give a sticky residue, which was partitioned between dichloromethane (60 mL) and a 0.1 N NaOH solution (20 mL). The organic phase was collected and concentrated, and the resulting solid was purified by column chromatography on silica gel to yield compound **1a** (1.65 g, 36%). White solid; m.p.: 144–146 °C; ^1^H-NMR (400 MHz, CDCl_3_) δ (ppm): 7.86 (1H, d, *J* = 7.6 Hz, PhH), 7.79 (1H, m, PhH), 7.14 (1H, t, *J* = 8.0 Hz, PhH), 5.15 (4H, m, CH), 4.28 (2H, m, CH), 3.93 (1H, d, *J* = 4.0 Hz, CH), 3.90 (3H, s, OCH_3_), 2.10 (3H, s, CH_3_), 2.07 (3H, s, CH_3_), 2.02 (6H, s, CH_3_); ESI-MS (*m/z*): 523 [M+Na]^+^; HRMS-ESI (*m/z*): calculated for C_22_H_26_FO_12_ [M+H]^+^: 501.14083; measured: 501.14078.

*Methyl 2,6-dimethoxy-4-((2S,3R,4S,5R,6R)-3,4,5-triacetoxy-6-(acetoxymethyl)-tetrahydro-2H-pyran-2-yloxy)benzoate* (**2a**). Compound **2a** was synthesized using a method similar to that described above for **1a**. Yield: 35%, white solid; m.p.: 121–123 °C; ^1^H-NMR (400 MHz, CDCl_3_) δ (ppm): 6.50 (2H, s, PhH), 5.28 (3H, m, CH), 5.15 (1H, d, *J* = 7.2 Hz, CH), 4.22 (1H, m, CH), 4.09 (1H, d, *J* = 8.4 Hz, CH), 3.89 (3H, s, OCH_3_), 3.85 (6H, s, OCH_3_), 3.69 (1H, m, CH), 2.04 (3H, s, CH_3_), 2.01 (9H, s, CH_3_); ESI-MS (*m/z*): 565 [M+Na]^+^; HRMS-ESI (*m/z*): calculated for C_24_H_31_O_14_ [M+H]^+^: 543.17138; measured: 543.17148. 

*Ethyl 3,5-dichloro-4-((2S,3R,4S,5R,6R)-3,4,5-triacetoxy-6-(acetoxymethyl)-tetrahydro-2H-pyran-2-yloxy)benzoate* (**3a**). Compound **3a** was synthesized using a method similar to that described above for **1a**. Yield: 32%, white solid; m.p.: 112–114 °C; ^1^H-NMR (400 MHz, CDCl_3_) δ (ppm): 7.93 (2H, s, PhH), 6.39 (1H, s, CH), 5.30 (3H, m, CH), 4.33 (3H, m, CH), 4.11 (2H, m, CH), 2.06 (3H, s, CH_3_), 2.03 (9H, s, CH_3_), 1.33 (3H, s, CH_3_); ESI-MS (*m/z*): 587 [M+Na]^+^; HRMS-ESI (*m/z*): calculated for C_22_H_25_Cl_2_O_12_ [M+H]^+^: 551.07231; measured: 551.07226. 

*Methyl 4-amino-3-((2S,3R,4S,5R,6R)-3,4,5-triacetoxy-6-(acetoxymethyl)-tetrahydro-2H-pyran-2-yloxy)benzoate* (**4a**). Compound **4a** was synthesized using a method similar to that described above for **1a**. Yield: 29%; brown solid; m.p.: 126–128 °C; ^1^H-NMR (400 MHz, CDCl_3_) δ (ppm): 7.84 (1H, d, *J* = 6.0 Hz, PhH), 7.77 (1H, m, PhH), 7.10 (1H, t, *J* = 8.8. Hz, PhH), 5.54 (1H, t, *J* = 8.4 Hz, CH), 5.44 (1H, d, *J* = 3.2 Hz, CH), 5.14 (1H, dd, *J* = 8.8 Hz, *J* = 3.2 Hz, CH), 5.02 (1H, d, *J* = 8.4 Hz, CH), 4.21 (2H, m, CH), 4.05 (1H, t, *J* = 6.4 Hz, CH), 3.90 (3H, s, OCH_3_), 3.74 (2H, s, NH_2_), 2.19 (3H, s, CH_3_), 2.10 (3H, s, CH_3_), 2.07 (3H, s, CH_3_), 2.00 (3H, s, CH_3_); ESI-MS (*m/z*): 520 [M+Na]^+^; HRMS-ESI (*m/z*): calculated for C_22_H_28_NO_12_ [M+H]^+^: 498.16115; measured: 498.16102. 

*Methyl 4-((2S,3R,4S,5R,6R)-3,4,5-triacetoxy-6-(acetoxymethyl)-tetrahydro-2H-pyran-2-yloxy)benzoate* (**5a**). Compound **5a** was synthesized using a method similar to that described above for **1a**. Yield: 30%; white solid; m.p.: 147–149 °C; ^1^H-NMR (400 MHz, CDCl_3_) δ (ppm): 8.00 (2H, d, *J* = 8.4 Hz, PhH), 7.00 (2H, d, *J* = 8.4 Hz, PhH), 5.53 (1H, d, *J* = 7.6 Hz, CH), 5.27 (2H, m, CH), 5.14 (1H, t, *J* = 6.4 Hz, CH), 5.02 (1H, d, *J* = 7.2 Hz, CH), 4.25 (4H, m, CH), 2.08 (3H, s, CH_3_), 2.05 (6H, s, CH_3_), 2.04 (3H, s, CH_3_), 1.38 (3H, s, CH_3_); ESI-MS (*m/z*): 519 [M+Na]^+^; HRMS-ESI (*m/z*): calculated for C_23_H_29_O_12_ [M+H]^+^: 497.16590; measured: 497.16594. 

*Methyl 3-fluoro-4-((2S,3R,4S,5R,6R)-3,4,5-triacetoxy-6-(acetoxymethyl)-tetrahydro-2H-pyran-2-yloxy)benzoate* (**7a**). Compound **7a** was synthesized using a method similar to that described above for **1a**. Yield: 31%; white solid; m.p.: 121–123 °C; ^1^H-NMR (500 MHz, CDCl_3_) δ (ppm): 7.83 (1H, t, *J* = 6.5 Hz, PhH), 6.90 (1H, dd, *J* = 10.5 Hz, *J* = 2.5 Hz, PhH), 6.83 (1H, t, *J* = 8.0 Hz, PhH), 5.57 (1H, d, *J* = 4.0 Hz, CH), 5.21 (4H, m, CH), 4.37 (2H, m, CH), 3.90 (3H, s, OCH_3_), 2.10 (6H, s, CH_3_), 2.06 (6H, s, CH_3_); ESI-MS (*m/z*): 523 [M+Na]^+^; HRMS-ESI (*m/z*): calculated for C_22_H_26_FO_12_ [M+H]^+^: 501.14083; measured: 501.14091. 

*Ethyl 4-fluoro-2-((2S,3R,4S,5R,6R)-3,4,5-triacetoxy-6-(acetoxymethyl)-tetrahydro-2H-pyran-2-yloxy)-benzoate* (**8a**). Compound **8a** was synthesized using a method similar to that described above for **1a**. Yield: 30%; white solid; m.p.: 136–138 °C; ^1^H-NMR (500 MHz, CDCl_3_) δ (ppm): 7.80 (1H, t, *J* = 7.5 Hz, PhH), 6.95 (1H, dd, *J* = 8.0 Hz *, J* = 2.0 Hz, PhH), 6.84 (1H, m, PhH), 5.92 (1H, t, *J* = 9.0 Hz, CH), 5.48 (1H, d, *J* = 3.5 Hz, CH), 5.08 (2H, m, CH), 4.17 (3H, m, CH), 3.85 (3H, s, OCH_3_), 2.16 (3H, s, CH_3_), 2.09 (3H, s, CH_3_), 2.05 (3H, s, CH_3_), 2.01 (3H, s, CH_3_); ESI-MS (*m/z*): 523 [M+Na]^+^; HRMS-ESI (*m/z*): calculated for C_22_H_26_FO_12_ [M+H]^+^: 501.14083; measured: 501.14082.

*Methyl 2-fluoro-6-((2S,3R,4S,5R,6R)-3,4,5-triacetoxy-6-(acetoxymethyl)-tetrahydro-2H-pyran-2-yloxy)benzoate* (**9a**). Compound **9a** was synthesized using a method similar to that described above for **1a**. Yield: 35%; white solid; m.p.: 130–132 °C; ^1^H-NMR (500 MHz, CDCl_3_) δ (ppm): 7.31 (1H, q, *J* = 8.5 Hz, PhH), 6.90 (1H, d, *J* = 8.5 Hz, PhH), 6.86 (1H, t, *J* = 8.0 Hz, PhH), 5.27 (2H, m, CH), 5.18 (1H, t, *J* = 6.5 Hz, CH), 5.05 (1H, d, *J* = 7.5 Hz, CH), 4.25 (3H, m, CH), 3.89 (3H, s, OCH_3_), 2.11 (3H, s, CH_3_), 2.08 (3H, s, CH_3_), 2.05 (3H, s, CH_3_), 2.02 (3H, s, CH_3_); ESI-MS (*m/z*): 523 [M+Na]^+^; HRMS-ESI (*m/z*): calculated for C_22_H_26_FO_12_ [M+H]^+^: 501.14083; measured: 501.14077.

*(2R,3R,4S,5R,6S)-2-(Acetoxymethyl)-6-(3-fluoro-2-methylphenoxy)-tetrahydro-2H-pyran-3,4,5-triyl triacetate* (**10a**). Compound **10a** was synthesized using a method similar to that described above for **1a**. Yield: 33%; white solid; m.p.: 127–129 °C; ^1^H-NMR (500 MHz, CDCl_3_) δ (ppm): 7.62 (1H, m, PhH), 7.21 (1H, m, PhH), 6.90 (1H, dd, *J* = 9.5 Hz, *J* = 3.5 Hz, PhH), 6.33 (1H, d, *J* = 3.5 Hz, CH), 5.72 (1H, d, *J* = 8.0 Hz, CH), 5.50 (2H, m, CH), 4.57 (1H, t, *J* = 7.0 Hz, CH), 4.22 (2H, m, CH), 2.40 (3H, s, CH_3_), 2.07 (6H, s, CH_3_), 2.02 (6H, s, CH_3_); ESI-MS (*m/z*): 479 [M+Na]^+^; HRMS-ESI (*m/z*): calculated for C_21_H_26_FO_10_ [M+H]^+^: 457.15100; measured: 457.15095. 

*(2R,3R,4S,5R,6S)-2-(Acetoxymethyl)-6-(pyridin-4-yloxy)-tetrahydro-2H-pyran-3,4,5-triyl triacetate* (**11a**). Compound **11a** was synthesized using a method similar to that described above for **1a**. Yield: 27%; colorless solid; m.p.: 132–134 °C; ^1^H-NMR (500 MHz, CDCl_3_) δ (ppm): 8.30 (2H, d, *J* = 9.0 Hz, PhH), 6.93 (2H, d, *J* = 9.0 Hz, PhH), 5.50 (1H, dd, *J* = 10.5 Hz, *J* = 3.0 Hz, CH), 5.18 (1H, d, *J* = 3.0 Hz, CH), 5.05 (1H, t, *J* = 7.0 Hz, CH), 4.15 (4H, m, CH), 2.08 (3H, s, CH_3_), 2.04 (3H, s, CH_3_), 2.02 (6H, s, CH_3_); ESI-MS (*m/z*): 448 [M+Na]^+^; HRMS-ESI (*m/z*): calculated for C_19_H_24_NO_10_ [M+H]^+^: 426.14002; measured: 426.14006. 

*(2R,3R,4S,5R,6S)-2-(Acetoxymethyl)-6-(3-fluoropyridin-2-yloxy)-tetrahydro-2H-pyran-3,4,5-triyl triacetate*(**12a**). Compound **12a** was synthesized using a method similar to that described above for **1a**. Yield: 31%; pale yellow solid; m.p.: 140–142 °C; ^1^H-NMR (500 MHz, CDCl_3_) δ (ppm): 7.92 (1H, d, *J* = 5.0 Hz, PhH), 7.39 (1H, t, *J* = 8.5 Hz, PhH), 6.99 (1H, m, PhH), 6.18 (1H, d, *J* = 7.5 Hz, CH), 5.35 (2H, m, CH), 5.24 (1H, t, *J* = 9.5 Hz, CH), 4.30 (1H, dd, *J* = 12.5 Hz, *J* = 4.5 Hz, CH), 4.14 (1H, d, *J* = 12.5 Hz, CH), 3.94 (1H, m, CH), 2.10 (3H, s, CH_3_), 2.07 (3H, s, CH_3_), 2.04 (3H, s, CH_3_), 2.02 (3H, s, CH_3_); ESI-MS (*m/z*): 466 [M+Na]^+^; HRMS-ESI (*m/z*): calculated for C_19_H_23_FNO_10_ [M+H]^+^: 444.13060; measured: 444.13051. 

*(2R,3R,4S,5R,6S)-2-(Acetoxymethyl)-6-(5-(acetoxymethyl)-2-fluorophenoxy)-tetrahydro-2H-pyran-3,4,5-triyl triacetate* (**13a**). Compound **1a** (500 mg, 1 mmol) was added to THF (25 mL), and solid LiAlH_4_ was added in a portion-wise manner to the resulting solution. Upon completion of the addition, the mixture was stirred at 66 °C for 3 h before being cooled to ambient temperature and quenched by the dropwise addition of CH_3_OH (10 mL). The reaction mixture was then stirred at room temperature for 10 min and concentrated to dryness under vacuum. The resulting residue was then dissolved in a mixture of acetic anhydride (20 mL) and pyridine (1 mL). The reaction mixture was stirred at 80 °C for 4 h and then concentrated to dryness *in vacuo*. The residue was added to ice water and stirred until precipitation occurred. The mixture was then filtered, and the filter-cake was collected and purified by flash chromatography over silica gel, affording the title compound (401 mg, 78%). White solid; m.p.: 125–127 °C; ^1^H-NMR (500 MHz, CDCl_3_) δ (ppm): 7.80 (1H, d, *J* = 8.0 Hz, PhH), 7.74 (1H, m, PhH), 7.10 (1H, t, *J* = 8.5 Hz, PhH), 5.89 (1H, d, *J* = 6.5 Hz, CH), 5.30 (1H, dd, *J* = 10.5,4.0 Hz, CH), 5.07 (1H, t, *J* = 11.0 Hz, CH), 5.19 (2H, s, CH), 4.36 (1H, m, CH), 4.14 (3H, m, CH), 2.11 (3H, s, CH_3_), 2.08 (6H, s, CH_3_), 2.06 (3H, s, CH_3_), 2.04 (3H, s, CH_3_); ESI-MS (*m/z*): 537 [M+Na]^+^; HRMS-ESI (*m/z*): calculated for C_23_H_28_FO_12_ [M+H]^+^: 515.15648; measured: 515.15652. 

*(2S,3R,4S,5S,6R)-2-(2-Fluoro-5-(hydroxymethyl)phenoxy)-6-(hydroxymethyl)-tetrahydro-2H-pyran-3,4,5-triol* (**14a**). To a solution of **13a** (510 mg, 1 mmol) in CH_3_OH (10 mL) was added a 0.2 N solution of CH_3_ONa in CH_3_OH, and the resulting mixture was stirred at room temperature for 1 h and then allowed to stand at ambient temperature overnight. The reaction mixture was filtered, and the filter cake was collected and recrystallized from anhydrous ethanol to afford the title compound (280 mg, 90%). White solid; m.p.: 134–136 °C; ^1^H-NMR (500 MHz, CDCl_3_) δ (ppm): 7.15 (1H, t, *J* = 9.5 Hz, PhH), 6.95 (1H, dd, *J* = 9.5 Hz, *J* = 4.0 Hz, PhH), 6.91 (1H, m, PhH), 5.60 (1H, s, CH), 4.66 (2H, s, CH), 4.20 (1H, s, CH), 3.47 (5H, m, CH), 2.76 (1H, s, OH), 2.16 (1H, s, OH), 1.78 (2H, s, OH), 0.99 (1H, s, OH); ESI-MS (*m/z*): 327 [M+Na]^+^; HRMS-ESI (*m/z*): calculated for C_13_H_18_FO_7_ [M+H]^+^: 305.10366; measured: 305.10362. 

*Methyl 4-fluoro-3-((2S,3R,4S,5S,6R)-3,4,5-triacetoxy-6-(acetoxymethyl)-tetrahydro-2H-pyran-2-yloxy)benzoate* (**1b**). Compound **1b** was synthesized using a method similar to that described above for **1a**. Yield: 38%; white solid; m.p.: 143–145 °C; ^1^H-NMR (500 MHz, CDCl_3_) δ (ppm): 7.88 (1H, d, *J* = 6.5 Hz, PhH), 7.78 (1H, m, PhH), 7.14 (1H, t, *J* = 9.0 Hz, PhH), 5.53 (1H, t, *J* = 8.5 Hz, CH), 5.46 (1H, d, *J* = 3.0 Hz, CH), 5.11 (1H, dd, *J* = 10.0, 3.0 Hz, CH), 5.01 (1H, d, *J* = 8.0 Hz, CH), 4.20 (2H, m, CH), 4.07 (1H, t, *J* = 6.5 Hz, CH), 3.90 (3H, s, OCH_3_), 2.19 (3H, s, CH_3_), 2.10 (3H, s, CH_3_), 2.07 (3H, s, CH_3_), 2.00 (3H, s, CH_3_); ESI-MS (*m/z*): 523 [M+Na]^+^; HRMS-ESI (*m/z*): calculated for C_22_H_26_FO_12_ [M+H]^+^: 501.14083; measured: 501.14086.

*Methyl 3-((2S,3R,4S,5S,6R)-3,4,5-triacetoxy-6-(acetoxymethyl)-tetrahydro-2H-pyran-2-yloxy)benzoate* (**6b**). Compound **6b** was synthesized using a method similar to that described above for **1a**. Yield: 36%; white solid; m.p.: 154–156 °C; ^1^H-NMR (500 MHz, CDCl_3_) δ (ppm): 7.75 (1H, d, *J* = 8.0 Hz, PhH), 7.67 (1H, s, PhH), 7.37 (1H, t, *J* = 8.0 Hz, PhH), 7.20 (1H, dd, *J* = 8.0, 2.5 Hz, CH), 5.50 (2H, m, CH), 5.12 (2H, m, CH), 4.16 (3H, m, CH), 3.91 (3H, s, OCH_3_), 2.19 (3H, s, CH_3_), 2.07 (6H, s, CH_3_), 2.00 (3H, s, CH_3_); ESI-MS (*m/z*): 505 [M+Na]^+^; HRMS-ESI (*m/z*): calculated for C_22_H_27_O_12_ [M+H]^+^: 483.15025; measured: 483.15030. 

*Methyl 3-fluoro-4-((2S,3R,4S,5S,6R)-3,4,5-triacetoxy-6-(acetoxymethyl)-tetrahydro-2H-pyran-2-yloxy)benzoate* (**7b**). Compound **7b** was synthesized using a method similar to that described above for **1a**. Yield: 32%; white solid; m.p.: 121–123 °C; ^1^H-NMR (500 MHz, CDCl_3_) δ (ppm): 7.77 (2H, m, PhH), 7.21 (1H, t, *J* = 8.5 Hz, PhH), 5.52 (1H, t, *J* = 8.0 Hz, CH), 5.45 (1H, d, *J* = 3.0 Hz, CH), 5.11 (1H, dd, *J* = 10.5, 3.0 Hz, CH), 5.01 (1H, d, *J* = 7.5 Hz, CH), 4.20 (2H, m, CH), 4.05 (1H, m, CH), 3.90 (3H, s, OCH_3_), 2.15 (3H, s, CH_3_), 2.08 (3H, s, CH_3_), 2.03 (3H, s, CH_3_), 1.99 (3H, s, CH_3_); ESI-MS (*m/z*): 523 [M+Na]^+^; HRMS-ESI (*m/z*): calculated for C_22_H_26_FO_12_ [M+H]^+^: 501.14083; measured: 501.14076. 

*Methyl 4-fluoro-2-((2S,3R,4S,5S,6R)-3,4,5-triacetoxy-6-(acetoxymethyl)-tetrahydro-2H-pyran-2-yloxy)benzoate* (**8b**). Compound **8b** was synthesized using a method similar to that described above for **1a**. Yield: 30%; white solid; m.p.: 136–138 °C; ^1^H-NMR (500 MHz, CDCl_3_) δ (ppm): 7.83 (1H, t, *J* = 7.0 Hz, PhH), 6.92 (1H, dd, *J* = 8.5, 2.0 Hz, PhH), 6.83 (1H, m, PhH), 5.60 (1H, t, *J* = 8.0 Hz, CH), 5.47 (1H, d, *J* = 3.0 Hz, CH), 5.11 (1H, dd, *J* = 10.5, 3.5 Hz, CH), 5.04 (1H, d, *J* = 8.0 Hz, CH), 4.17 (3H, m, CH), 3.85 (3H, s, OCH_3_), 2.18 (3H, s, CH_3_), 2.08 (3H, s, CH_3_), 2.06 (3H, s, CH_3_), 2.02 (3H, s, CH_3_); ESI-MS (*m/z*): 523 [M+Na]^+^; HRMS-ESI (*m/z*): calculated for C_22_H_26_FO_12_ [M+H]^+^: 501.14083; measured: 501.14074. 

*Methyl 2-fluoro-6-((2S,3R,4S,5S,6R)-3,4,5-triacetoxy-6-(acetoxymethyl)-tetrahydro-2H-pyran-2-yloxy)benzoate* (**9b**). Compound **9b** was synthesized using a method similar to that described above for **1a**. Yield: 35%; white solid; m.p.: 130–132 °C; ^1^H-NMR (500 MHz, CDCl_3_) δ (ppm): 7.31 (1H, q, *J* = 8.5 Hz, PhH), 6.92 (1H, d, *J* = 8.0 Hz, PhH), 6.84 (1H, t, *J* = 8.5 Hz, PhH), 5.47 (2H, m, CH), 5.07 (1H, dd, *J* = 10.5 Hz, *J* = 3.0 Hz, CH), 5.00 (1H, d, *J* = 8.0 Hz, CH), 4.16 (3H, m, CH), 3.90 (3H, s, OCH_3_), 2.21 (3H, s, CH_3_), 2.12 (3H, s, CH_3_), 2.06 (3H, s, CH_3_), 2.03 (3H, s, CH_3_); ESI-MS (*m/z*): 523 [M+Na]^+^; HRMS-ESI (*m/z*): calculated for C_22_H_26_FO_12_ [M+H]^+^: 501.14083; measured: 501.14077. 

*(2R,3S,4S,5R,6S)-2-(Acetoxymethyl)-6-(3-fluoro-2-methylphenoxy)-tetrahydro-2H-pyran-3,4,5-triyl triacetate* (**10b**). Compound **10b** was synthesized using a method similar to that described above for **1a**. Yield: 36%; white solid; m.p.: 127–129 °C; ^1^H-NMR (500 MHz, CDCl_3_) δ (ppm): 7.60 (1H, m, PhH), 7.20 (1H, m, PhH), 6.93 (1H, dd, *J* = 9.5, 3.5 Hz, PhH), 6.32 (1H, d, *J* = 3.5 Hz, CH), 5.74 (1H, d, *J* = 8.0 Hz, CH), 5.50 (2H, m, CH), 4.54 (1H, t, *J* = 7.0 Hz, CH), 4.24 (2H, m, CH), 2.40 (3H, s, CH_3_), 2.09 (3H, s, CH_3_), 2.07 (3H, s, CH_3_), 2.04 (3H, s, CH_3_), 2.02 (3H, s, CH_3_); ESI-MS (*m/z*): 479 [M+Na]^+^; HRMS-ESI (*m/z*): calculated for C_21_H_26_FO_10_ [M+H]^+^: 457.15100; measured: 457.15098.

### 3.3. Antiviral Assays

MDCK cells, influenza A (A/Hanfang/359/95, H3N2), influenza A (A/FM/1/47, H1N1) and influenza B (B/Jifang/13/97) were obtained from the Institute of Virology of the Chinese Academy of Preventive Medicine (Beijing, China).

#### 3.3.1. Cytotoxicity Assay

The cytotoxicities of the compounds in the presence of MDCK cells were monitored by CPE. MDCK cells (2.5 × 10^4^/well) were plated into a 96-well plate. After 24 h, the monolayer cells were incubated in the presence of a variety of different concentrations of the test compounds. After 48 h of culture at 37 °C and under a 5% CO_2_ atmosphere in a carbon dioxide incubator, the cells were monitored by CPE. The median toxic concentrations (TC_50_) were determined using a Reed and Muench calculation.

#### 3.3.2. Anti-Influenza Assays

Confluent MDCK cells grown in 96-well microplates were infected with 100 median tissue culture infective dose units (100TCID_50_) of the test strains. Following 2 h of adsorption at 37 °C, the monolayers were washed with phosphate-buffered saline (PBS) and incubated at 37 °C in the maintenance medium either in the presence or in the absence of different concentrations of the test compounds. The viral CPE was observed when the viral control group reached 4, and the antiviral activities (IC_50_) of the bisaryl amide derivatives were determined using a Reed and Muench calculation. The SI value was calculated from TC_50_/IC_50_.

### 3.4. Antiviral Assays *in Vivo*

The effect of compound **1a** on influenza A/FM/1/47-MA (H1N1) virus-infected mice was evaluated during the course of the current study, with ribavirin being used as a reference standard. Mice (female, 5–6 weeks of age and 14–16 grams in weight) were purchased from the National Institute for the Control of Pharmaceutical and Biological Products (Beijing, China).

Ether-anesthetized mice were infected intranasally with a 50 times LD_50_ dose of mouse-adapted influenza A/FM/1/47-MA (H1N1) virus. Compound **1a**, prepared as a suspension in 5% tween-80, or 5% tween-80 alone (vehicle group), was administered intragastrically twice daily for seven days, with the dose regimen being initiated one day prior to viral infection. The 10 mice involved in each group were observed twice daily for 14 days during the experiment for the occurrence of death. The mean day-to-death and survival times were then calculated (Table 3).

In a separate study, all eight of the mice involved in each group were killed when deaths began to occur in the virus control group (day 6 post-virus exposure), and their lungs were removed aseptically. Visual evidence of lung consolidation was scored on a blind basis. Lung scores were based on the percentage of the lung displaying signs of consolidation, with 0 being normal and 4 being indicative of 100% consolidation. Lung consolidation scores were evaluated using ranked sum analysis (Table 3).

### 3.5. Mechanism Studies

#### 3.5.1. Hemagglutination Inhibition Assay

Hemagglutination inhibition (HI) assay was employed to evaluate the effect of a compound on virus adsorption to target cells according to the reference with slight modification. Briefly, compound in PBS was mixed with an equal volume of influenza virus that was serially diluted by two-fold in PBS. After 30 min incubation at 4 °C, 100 µL of compound-virus preparation was mixed with an equal volume of 1% chicken erythrocyte suspension in PBS in a U bottom 96-well plate. The mixture was incubated for 1 h at room temperature before observing erythrocyte aggregation on the plate.

#### 3.5.2. Neuraminidase Inhibition Assay

Neuraminidase inhibition assay were evaluated for *in vitro* inhibitory actions using the method according to the reference with slight modification. This spectrofluorometric assay (excitation wavelength: 355 nm and emission wavelength: 460 nm) uses 2′-(4-methylumbelliferyl)-*α*-d- acetylneuraminic acid (MUNANA) as the enzyme substrate, which is converted to a fluorescent product upon cleavage by NA. The intensity of fluorescence can reflect the activity of NA sensitively.

The enzyme reaction system included 10 μL sample, 30 μL enzyme and 60 μL substrate buffer mix (30 μm MUNAN, 33 mm MES buffer (pH 3.5), 4 mm CaCl_2_, DDW). The terminal volume was 100 μL. Two-fold serially diluted compound samples and 30 μL enzyme incubated 1 hour at room temperature, and 60 μL substrate buffer mix was added. After 15 min at 37 °C, 100 μL of 14 mm NaOH in 83% ethanol was added to the reaction mixture to terminate the reaction. The intensity of the fluorescence was quantitated in fluorescence read on Enspire (Perkin Elmer, Waltham, MA, USA) (excitation, 355 nm; emission, 460 nm), and substrate blanks were subtracted from the sample readings. The IC_50_ was calculated by plotting percent inhibition *versus* the inhibitor concentration, and determination of each point was performed in duplicate.

#### 3.5.3. M2 Protein Expression Inhibition Assay

The effect of compounds on M2 protein expression level were detected by immunoboltting assay. The immunoboltting experiment was employed according to the reference with slight modification.MDCK cells were infected with influenza virus A/Wuhan/359/95 at an 300TCID_50_, and treated with the indicated concentration of compounds. 24 hours post infection, the cells were lysed with lysis buffer (1% Triton, 150 mM NaCl, 20 mM HEPES pH7.5, 10% Glycerol, 1 mM EDTA) with protease inhibitor cocktail (Roche, Indianapolis, IN, USA). The lysates were then performed to immunoblotting with M2 andβ-actin antibodies (Influenza A m2 Antibody from Santa Cruz Biotechnology Inc. (Santa Cruz, CA, USA) and β-Actin Mouse mAb from Cell Signaling Technology Inc. (Danvers, MA, USA). Protein was detected using Immobilon Western Chemiluminescent HRP Substrate (Millipore, Inc., Billerica, MA, USA) with ChemiDoc XRS and Image Acquisition (BioRad, Inc., Hercules, CA, USA). Most studies of the activity and drug sensitivity of A/M2 channels have employed electrophysiology techniques, but since establishment of this technology takes time, we just detected M2 protein expression levels.

## 4. Conclusions

Gastrodin is a traditional Chinese herbal medicine that has been used clinically in China for thousands of years, mainly for the treatment of neurasthenia. To the best of our knowledge, there have been no reports documenting the use of gastrodin or any of its derivatives as antiviral agents. In our study, a novel series of gastrodin analogues was found to exhibit potent anti-influenza activity. A series of substituted aryl glycoside compounds were synthesized and evaluated for their anti-influenza virus activities, and preliminary structure-activity relationships (SAR) were analyzed.

A total of 15 novel glycoside compounds were synthesized and screened for their antiviral activity against influenza A virus, among which the most potent inhibitor **1a** exhibited moderate inhibitory activity against the virus. Herein, we also report the first total synthesis of **1a** and its associated *in vivo* anti-influenza data. In this experiment, a dose regimen of 80 mg/kg/day of compound **1a** (oral administration) yielded a significant increase in the survival numbers and prolonged the mean survival time of the animals tested.

Furthermore, the data were analyzed to produce a preliminary understanding of the structure-activity relationships. Seven compounds exhibited clear antiviral activity against the tested strain of the influenza A virus. The IC_50_ and SI values of the most effective compound, **12a**, against the influenza A (A/FM/1/47(H1N1)) virus were 25.24 μM and 13.9, respectively. Unfortunately, the SI values for all the compounds tested were much lower than those of the reference compounds. Remarkably, compounds **1a**, **2a** and **10b** also displayed antiviral activity against the oseltamivir-null strain of influenza B. In addition, there was an increasing need for the development of antiviral along with novel antiviral mechanism. Compound **1a** had a different mechanism of action from oseltamivir and ribavirin against influenza virus, so future research may be more meaningful with this result.

Undoubtedly, further exploration will be needed to produce more potent compounds with higher SI values and to deduce the detailed mechanism of action of this novel class of glycosides compounds against the influenza virus. Besides, since compound **1a** has been demonstrated that it has no interaction with NA or M2, it probably has novel targets which might be an important breakthrough in viral study.
